# Implementation of an Intervention Plan for Emotional Development in People with Down Syndrome

**DOI:** 10.3390/ijerph18094763

**Published:** 2021-04-29

**Authors:** Macarena Castellary-López, Juan Rafael Muñoz Muñoz, Victoria Figueredo-Canosa, Luis Ortiz-Jiménez

**Affiliations:** Departament of Education, Faculty of Education, University of Almeria, 04120 Almeria, Spain; mcl142@ual.es (M.C.-L.); jrmunoz@ual.es (J.R.M.M.); vfc310@ual.es (V.F.-C.)

**Keywords:** music, emotions, Down syndrome

## Abstract

The importance of music, as well as the different and diverse possibilities that it offers, favors the emotional development of any person. This research is based on the development and application of a set of activities, whose transversal axis is the use of music, to favor and promote the emotional development of people with Down syndrome. This application of activities was developed with a group of eight participants, between the ages of twenty and forty-five years old. Additionally, under a total duration of eight working sessions. In these sessions, listening, vocal, instrumental, and movement activities were developed. For each of the emotions worked on; joy, fear, anger, sadness, calm, and love, a story and a song from the story were selected for each one of them. The methodology used was qualitative, using program evaluation. For this purpose, on the one hand, the data obtained during the different sessions were analyzed, and on the other hand, the data collected in the two discussion groups carried out were analyzed. Finally, the data obtained were organized into six categories: image recognition, observation of emotions, experience of emotions, identification of emotions, recognition of emotions, and finally, enjoyment of emotions. It could be seen that, after the sessions, there was a significant improvement in the different categories. However, in the categories of identification of emotions and recognition of emotions, the results were more favorable compared to the rest.

## 1. Introduction

The origin of emotional development goes back to 1983, with Gardner’s theory of multiple intelligences, where he identified interpersonal and intrapersonal intelligence. It was in 1990 when Mayer and Salovey established the five main competences; knowing one’s own emotions, the ability to control them, the ability to motivate oneself, to recognize other people’s emotions, and to control relationships [1]. Similarly, Mayer and Salovey established the three dimensions of emotional intelligence: perception, understanding, and regulation of emotions [2]. But what are emotions?

Emotions are responses that, for the most part, are innate to situations or events we have experienced, are happening, or imagine will happen. These present a subjective character, that is to say, they will be delimited by each person, nevertheless, almost all the people perceive and express emotions [3].

According to this, do we talk of emotional education or emotional development?

Emotional education, continuing with the idea of Vivas [4], is a process in which the perception, assimilation, understanding, regulation, and expression of emotions must be developed, while emotional development, continuing with that proposed by Bisquerra [5], is the process by which people acquire these capacities, so that both terms are related [6].

People with Down syndrome perceive and identify their own emotions, however, with respect to the emotions of others, they present a greater difficulty.

Next, the other main focus of the research, music education, will be dealt with.

### 1.1. Music and Emotions

Music is present in the lives of individuals from birth to the end of their days and has the ability to transmit and generate emotions, so it can be a resource that contributes to emotional development.

Music has different functions; these can be differentiated between teaching music, from a purely educational point of view, and the use of music for other purposes [7].

With respect to music and special educational needs, there have been different educators, such as Émile Jaques-Dalcroze, María Montessori, Edgar Willems, or Carl Orff among others [3], whose methodological trends have been used for students with special educational needs [8]

There are several authors, such as Lacárcel [9], Jauset [10], or Arañó [11] who defend the direct relationship between music and emotions.

In turn, as Castellary, Muñoz, and Ortiz [12] state, there are various authors who highlight the importance of using music with students with special educational needs, such as Lacárcel [13], Amusategui [14], or Prause-Weber [15].

On the one hand, these authors, Amusategui [14], Prause-Weber [15], and Lacárcel [13] emphasized the importance of using music with students with special educational needs, as, according to the authors, it can help to improve social interactions and communication.

On the other hand, they highlighted the importance of music as an artistic element, considering that the non-verbal component of music is fundamental for working with students with special educational needs.

All this leads us to consider what special educational needs are, as well as the relationship between these and emotions, and more specifically, the relationship between Down syndrome and emotions.

### 1.2. Down Syndrome and Emotions

Education for All was born in 1990, at a meeting held by UNESCO [16], where inclusive education was defended as an educational model.

There are different conceptions of the term special educational needs. One approach is that proposed by the National Resource Centre for Special Education, collected by Gómez [17], which focuses on the difficulties presented by the individual. Another, like the one collected by Sánchez and Torres [18], focuses on the attention and type of response that the different institutions should provide.

With regard to the classifications on special educational needs, there are different types of classifications, such as that collected by the World Health Organization [19], which focuses on a bio-psychosocial model, or that collected in the Psychopedagogical Encyclopaedia of Special Educational Needs [20].

Within the set of special educational needs, attention will be focused on Down syndrome.

Down syndrome is a chromosomal abnormality, where cells carry 47 chromosomes instead of 46. There are different types, depending on the number of cells that carry 47 chromosomes. However, in all cases, there is an extra chromosome of the pair 21 [21].

Its main characteristics are hypotonia, less coordination, disappearance of reflexes, affected auditory and visual development. With regard to cognitive and intellectual development, these are affected. On the other hand, language is late. Regarding the emotional development, they have a life rich in emotions, however, they present certain difficulties with the emotions of others, as well as in the expression of the same [22].

With regard to the difficulties that people with Down syndrome may have in recognizing emotions, Roch et al. [23] reported that they may have difficulty in recognizing facial expression in emotions such as fear. Similarly, they pointed out that, in general, with regard to negative emotions, the degree of difficulty in recognizing them is greater.

Finally, due to the importance of the recognition of emotions and knowledge of these, as well as the use of music as a resource for working with people with special educational needs, the main objective of this research was to verify how the use of music improves the emotional development of students with Down syndrome.

The results obtained showed a greater recognition of emotions as well as their identification. At the same time, we were able to confirm how the use of music in most of the activities carried out positively favored the emotional development of students with Down syndrome.

## 2. Materials and Methods

On a methodological level, in view of what was set out in the theoretical framework, a series of questions were raised: Does the use of songs serve to experience and raise awareness of basic emotions in participants with Down syndrome? Does the use of songs contribute to the identification of basic emotions in students with Down syndrome? Does the use of songs contribute to improving the control of emotions in participants with Down syndrome?

### 2.1. Aims

After the previous questions, a main objective was set; to verify how the use of music improves the emotional development of participants with Down syndrome.

This objective has been translated into various sub-objectives:

- To use the song as a means to observe, experience, and become aware of basic emotions in students with Down syndrome.
- To observe how the use of songs contributes to the identification of basic emotions in students with Down syndrome.
- To see how the use of songs contributes to improving the control of emotions in students with Down syndrome.

From the methodological point of view, it is necessary to indicate that the research is qualitative, developing a program evaluation.

### 2.2. Data-Collecting

The following instruments were used for data collection: observation sheets, field diary, and discussion groups.

- Observation sheets: a rubric was developed in order to collect information directly and immediately.
- Field diary: it facilitated the collection of data that could not be indicated in the rubric such as facial expressions or gestures of the participants.
- Focus groups were formed by the therapists and took place in two stages. The first focus group was before the intervention and the second focus group took place after the end of the intervention. In this way, it was possible to compare the data obtained and to find out if there were significant differences.

As for the participants, the selection of them was intentional, thus being able to obtain as much information and knowledge as possible about the object of study.

- Initially, families and/or guardians were to participate, but due to external causes, this could not be possible, so the participants went.
- Therapists, because they are the people responsible for working with them in the different training programs. The discussion groups were developed with them, both the discussion group prior to the intervention plan and the final discussion group.
- A total of 8 participants; 5 men and 3 women, between the ages of 20 and 45. For the selection of the 8 participants, it was decided that there would be no limitation in terms of characteristics or needs that they could present, as the activities would be adapted to the group.

The timeframe for this investigation was four weeks long. In the first week, the first group of discussion was developed with the therapists and two working sessions took place. During the following two weeks, three working sessions took place every other day. Finally, in the fourth week, the second discussion group with the therapists took place.

### 2.3. Triangulation

In order to triangulate the data collected, the following instruments were used to collect information: observation sheets, logbook or field diary (in order to be able to collect all those situations that could not be recorded on the observation sheets), review of documents prepared by the participants and two discussion groups, the first of which was held prior to the intervention plan and the second after it was completed, thus enabling the data obtained to be compared. The data analysis was structured in four blocks:

- Results of the observations.
- Results of the discussion groups.
- Results of the field diary.
- Discussion of the results.

This research was approved by the Bioethics Committee of the University of Almeria (Ref. UALBIO 2019/039). That it is defendant for reserve EDU2016-75574-P and for my doctoral thesis.

### 2.4. Data Analysis

A total of 6 categories were established for the data analysis and a scale of appreciation was used with the values: a lot, quite a lot, a little, and nothing. Each of the values was attributed a numerical value, being: 3, a lot; 2, quite a lot; 1, a little; 0, nothing.

The categories developed were:

- Image recognition.
- Observation of the emotions.
- Experiencing emotions.
- Identifying emotions.
- Recognition of emotions.
- Enjoy the emotions.

### 2.5. Intervention Plan

The intervention plan prepared included a total of eight working sessions, each session lasting 55 min.

In the first session, all the emotions were worked on in order to know which emotions they knew and/or recognized. In each of the following six sessions, an emotion was worked on, these were joy, fear, sadness, anger, calm, and love. Finally, in the eighth session, the whole set of emotions was worked on again. In this way, the data collected in the first and eighth session could be compared.

For each of the emotions, a piece of music was selected, as well as a story and a song from the story.

Table 1 Collected from Castellary [3].

The activities developed were dance, drawing, storytelling, vocal expression, assembly, instrumental accompaniment, or free movement. The model developed in common with the group of sessions is presented below.

Table 2 Recollected from Castellary [3].

## 3. Results

The results obtained from the research are presented by categories. In each of them, we will find a graph showing the evolution of the category throughout the eight work sessions, except in the first category, where the graph shows the differences between the first and the eighth session.

### 3.1. Image Recognition

In the first category, image recognition, the greatest image recognition, on a general level, was in the emotions joy, fear, and calm. While the lowest image recognition was in the emotions anger, sadness, and love.

**Figure 1:**
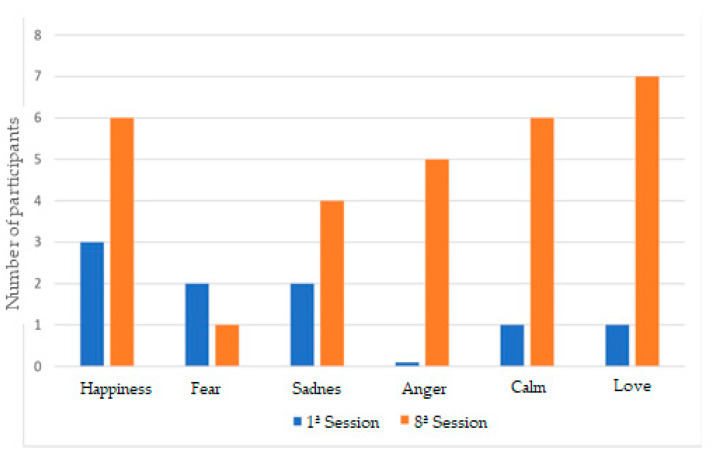
1st Category; Image recognition.

As can be seen in Figure 1, in the first session, we could see how the images recognized were; joy by three participants, sadness by two participants, anger by two participants, calm by one participant, and as for the emotion fear, the image was not recognized by any of the participants.

However, after the specific sessions for each of the emotions, it was possible to see how in the eighth session, the recognized images were; love for seven of the participants, joy for six of the participants, calm for six of the participants, fear for five of the participants, anger for four of the participants, and sadness for one of the participants.

These data are consistent with Ruiz’s approach [26], where he indicated that they “tend to confuse some positive emotions with negative emotions”.

One of the significant data were provided by the discussion groups. At first, therapists indicated that it was difficult for participants to recognize basic emotions, but after the working sessions, they indicated that participants were able to do so. In turn, this was also noted in the data obtained, as in the first session, the images were recognized by between one and three participants, while in the eighth session, they were recognized by between five and seven participants.

### 3.2. Observation of Emotions

**Figure A1:**
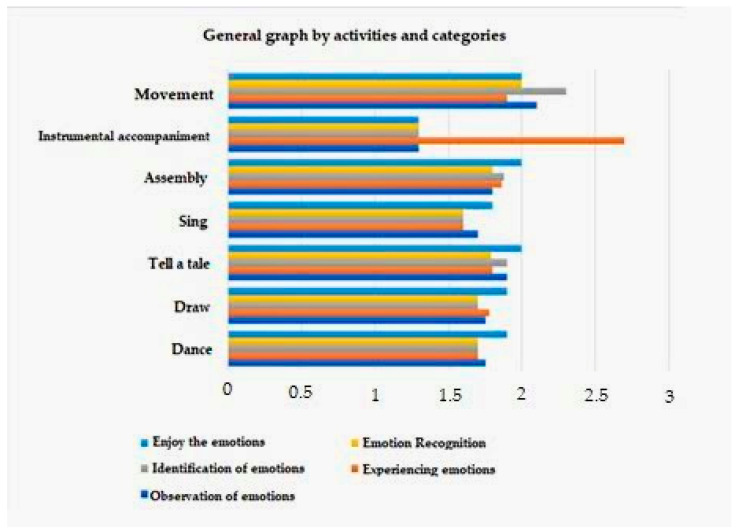
General graph by activities and categories.

We found on the one hand, that the activities most conducive to the development of observation were those of narration and movement, as can be seen in Figure A1 of the Appendix A.

**Figure 2:**
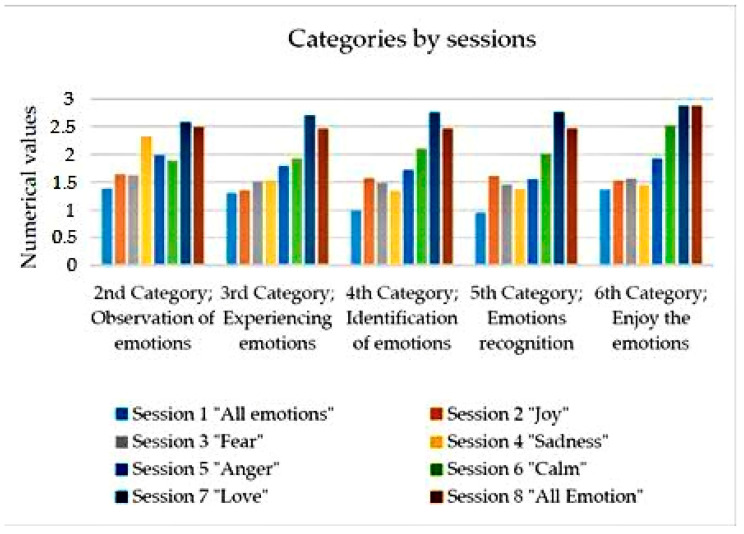
Categories by sessions.

On the other hand, the results obtained at a general level, as can be seen in Figure 2, showed a favorable trend of the results, since the more the sessions advanced, the results improved, going from four to five participants who observe the emotions quite a lot, to five of the participants who observe the emotions a lot.

On an individual level, it was possible to see how the improvement in results was evident the further the work sessions progressed. This is the case of participant 1 (P.), who if in the first two sessions, his observation of emotions was none, in the rest of the sessions, that is to say, six, his values increased almost until reaching a little observation of emotions. This could be due to his scarce participation or to the fact of his selective mutism.

At the same time, this participant showed greater attention and participation with the narrative activity, that is to say, this activity led to the values obtained being 1, little observation, as opposed to the rest of the activities as a whole, where the values were below 1.

On the other hand, an improvement in the results could be seen with participant 8 (N.). Although it is true that this participant showed little observation of emotions in the first session, from the second session onwards, his results improved until he achieved a considerable observation of emotions, continuing these results over four sessions. Finally, in the last two sessions, an improvement of the results could be appreciated, reaching a lot of observation of the emotions, so that the general improvement of the participants was more than significant.

With regard to the activities, those that were most appropriate for the participants were those of movement, assembly, dance, narration, vocal interpretation, drawing, and finally, instrumental accompaniment. Likewise, it is necessary to indicate that all the activities obtained values equal to or higher than 2, that is, they were quite favorable activities for the development of the observation of emotions in the participant 8 (N.).

### 3.3. Experiencing Emotions

In the third category, the experience of emotions, the number of activities that were most beneficial increased (Figure A1). These were: dance, drawing, storytelling, vocal interpretation, assembly, and movement.

As for the activities that most favored the experience, all of them are related to the story of the session, to the musical work or song of the story. This reaffirms the idea put forward by Mayer and Salovey, when they indicated that “literature is probably the first home of emotional intelligence. But so are art, music and theatre programmes” [27].

Focusing on the evolution per session of this category, it can be seen how there is an upward growth of the results. This growth occurred consecutively from the third session to the seventh. As it can be seen in Figure 2, the values obtained increased from 1.3 to 2.57, that is to say, the increase was 1.27, so that it went from a little experience of the emotions to a lot of experience.

**Figure A2:**
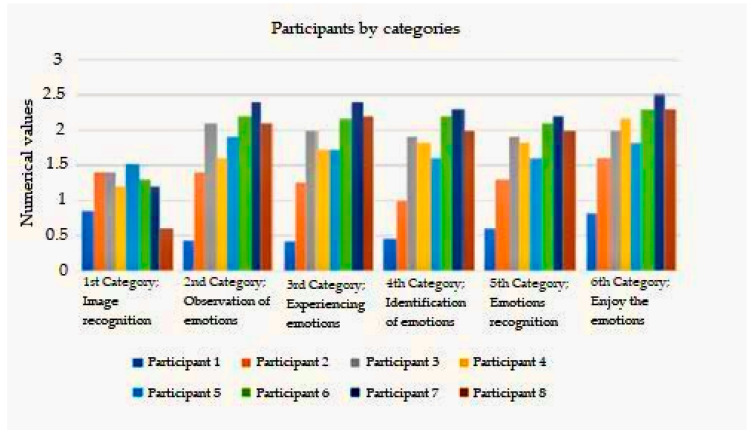
Participants by categories.

In this sense, it is worth mentioning participant 2 (V.), who, with respect to the experience of emotions, shows little experience in the first two sessions, but, from the fourth session onwards, achieved a considerable experience of emotions (Figure A2). Specifically, in the fourth session, he obtained values a little higher than 1, until reaching the value 2 in the seventh and eighth session.

On the other hand, the activities that most favured the development of this category in participant 2 were assembly, in the first place, and drawing. This comes to reaffirm the idea raised before about Mayer and Salovey [27], because, the activity of assembly was linked to the activity of narration, that is, literature, while the activity of drawing made use of the musical work selected for the own development of the activity.

Another significant data, referred to user 4 (M.), is the progress he presented, because although this user attended a total of five sessions, a favorable trend in his results with respect to this category could also be observed. This was confirmed after the first two sessions he attended, session 1 and 3, where the values obtained were between 0.6 and 1.35, that is, there was little experience. However, in the sixth session, it reached the value 2, and in the seventh session, the value 3, so it amounted to a quite first-hand experience, and finally, to a lot of first-hand experience of the emotions.

With regards to the activities that favored these results in participant 4 (M.), we find that firstly there are the activities of movement and drawing, followed by assembly, vocal interpretation, and narration. All of them had a considerable impact on the user due to the value reached, equal or superior to two.

### 3.4. Identification of Emotions

The fourth category, identification of emotions, showed that the activities most appropriate for its development were those of movement, narration, and assembly (Figure A1).

At the same time, focusing on the set of eight sessions developed, these show that, during the first session, the identification of the emotions was enough in the assembly activity, while, in the rest of the activities, there was little experience.

However, as the sessions progressed, it became clear that the data varied favorably, with the last two sessions showing a high degree of identification of emotions in six of the eight activities carried out by between 50% and 80% of the participants, with the other 50% and 20% of the participants corresponding to a high degree of identification.

If we focus on the evolution of the different sessions carried out, we can see a significant improvement in the data from the fourth session onwards, as can be seen in Figure 2. At the same time, these data show that, although in the first five sessions, there was a certain improvement within a little identification of the emotions, it was from the sixth session onwards that values close to 2 were reached, that is to say, a considerable identification of the emotions, reaching its maximum value in the seventh session, with a general value of more than 2.5.

This progress, however, was not the same for all the participating users, as participant 5 (J.) showed during the first five sessions, a little identification of emotions, however, within these first five sessions, there were important differences, for example, in the first two sessions his values were lower or equal to 1, while, in the following three sessions, these were 1.5. On the other hand, in the sixth session, it did show quite a lot of identification of the emotions, specifically, under the value 2. Its maximum identification of the emotions was found in the seventh session, specifically, it reached a lot of identification. Regarding the activities that favored quite a lot, the identification of emotions in user 5 (J.), they were those of movement and assembly. At the same time, although to a lesser extent, the activities that favored little, the identification was; dance, drawing, narration, vocal interpretation, and instrumental accompaniment. As it can be observed, the activities that favored quite a lot, the identification of emotions are related to the idea raised by Mayer and Salovey [27], where they indicated that both literature and art programs are the home of emotional intelligence.

### 3.5. Emotion Recognition

As for the fifth category, recognition of emotions. We found that, on the one hand, the activities that led to a greater recognition of emotions, on a general level, were firstly the activity of movement, followed by the activities of; assembly, narration, drawing, and dance (Figure A1).

With regard to participants 2 and 3, who communicated in a gestural way, it could be seen how, in the first sessions, their body posture was tense, while, from the fifth session onwards, their body posture was relaxed, even getting up from the chair to participate in movement and dance activities, as recorded in the field diary [3].

On the other hand, it is worth noting that while in the first sessions the recognition of emotions was between a little and a lot, in the last sessions, as shown in Figure 2, there was between quite a lot and a lot of recognition of emotions.

One of the participants to highlight was participant 6 (P. M.), who during the first session, showed little recognition of emotions, specifically with a value of 1. While in the following four sessions, he obtained quite a lot of recognition of emotions with a value equal to 2, however, in the last two sessions, sixth and seventh, the growth was quite significant as he presented a lot of recognition of emotions (Figure A2).

As for the activities that greatly favored the recognition of emotions by the participant 6 (P.M.), they were a large majority, specifically these were: movement, assembly, vocal interpretation, narration, drawing, and dance. The activity that led to little recognition of emotions was that of instrumental accompaniment. All these data lead us to reaffirm, on the one hand, the idea gathered from Mayer and Salovey [46], and on the other hand, that a large majority of the activities they favored are closely related to the selection of works and songs from stories.

### 3.6. Enjoy the Emotions

As far as the sixth category is concerned; enjoy the emotions. It can be seen how the activities that most favored enjoyment were those of movement, assembly, narration, drawing, and dance.

On the other hand, the level of enjoyment increased considerably from the sixth session, as can be seen in Figure 2, because although in that session, four of the participants enjoyed the emotions in the different activities carried out, in the seventh and eighth session, it was seven of the participants who enjoyed the emotions very much.

This can be seen with participants 2, 3, and 4, who in the first sessions, the level of enjoyment was low, while in the last three sessions, it increased to a high level, as can be seen in Table 3. It can be considered, therefore, that as the sessions were taking place, the degree of enjoyment was also increasing.

In the same way, participant 7 (J.L.) presented from the first session quite enjoyable emotions, specifically during the first three sessions. From the fifth session onwards, he showed a favorable increase in results, reaching value 3, i.e., a lot of enjoyment of the emotions. If we focus on the activities that were developed, it can be seen that six of the activities, in participant 7 (J.L.), favored quite a lot the enjoyment of emotions. At the same time, one of the activities, specifically the movement activity, greatly favored the enjoyment of emotions. This activity is highly linked to music, as the development of this depended on the use of the musical work selected for the session.

## 4. Discussion/Conclusions

With regard to the first category, image recognition, the most recognized images were joy, fear, and calm. The images related to emotions; anger, sadness, and love, whose recognition was lower. This is consistent with the approach of Ruiz [26] and Roch et al. [23], who indicated that they tend to have greater difficulty in recognizing negative emotions and that there is even confusion between positive and negative emotions.

On the other hand, Mayer and Salovey [27] stated the importance of narration and storytelling to improve the perception of emotions. This could be seen in category 3; Experiencing emotions, where one of the activities that most favored this category was storytelling.

Finally, as far as the conclusions are concerned, the data obtained have made it possible to observe how, initially, participants with Down syndrome had difficulty in recognizing and identifying emotions.

In turn, the songs and works selected, as well as the stories, have been fundamental to the development of each of the activities.

The most favorable results have been in the categories of recognition and identification of emotions. On the other hand, in each of the different categories, it has been possible to observe an improvement in the emotional development of participants with Down syndrome.

As for the activities, the one that has presented the most favorable values has been that of movement, specifically in four of the categories.

At the same time, in the activities of vocal interpretation, drawing, and dance, close to value 2, that is, quite a lot, within the scale of appreciation, they led to improvement in each of the categories developed. All these activities, movement, vocal interpretation, drawing, and dance have had as their fundamental axis the songs and works selected.

With regards to the materials, the results allow us to confirm that these have been favorable for the improvement of the emotional development of participants with Down syndrome.

Focusing on each of the categories, in general terms, there is a favorable trend in the values obtained.

However, some of the limitations of the study arose from the number of participants, with a total of eight, making it possible, as future research, to have a larger sample for the study. At the same time, another limitation of the research was the timing of the study, with eight working sessions, and although the results were significant, future research could focus on a larger number of sessions.

Finally, it is considered that, due to the presence and use of music in the vast majority of activities, as well as the results obtained in each one of them, music has facilitated and favored the improvement of emotional development in participants with Down syndrome.

## Figures and Tables

**Table 1:** Selection of musical works, stories, and songs.

Emotion	Musical Piece	Tale	Song
Happiness	Happy	El hada del Arco Iris	El hada del Arco Iris
Fear	BSO Tiburón	Jonás y el frigorífico miedoso	Jonás y el frigorífico miedoso
Sadness	Goodbye	Sara, la princesa especial	Sara, la princesa especial
Anger	María de la O	La rabieta de Julieta	La rabieta de Julieta
Calm	Canción de Cuna	Un puñado de besos	Un puñado de besos
Love	Claro de Luna	Doña Pescadilla	Doña Pescadilla
Nos despedimos		El monstruo de colores	El monstruo de los colores

**Table 2:** Activities developed in the different working sessions.

	Description	Organization	Methodology	Grouping	Place	Time	Recourses
Dance	Dance the song “Si los hombres han llegado hasta la luna” [24] Standing in front of the teacher, as they listen to the song, they will imitate her gestures and movements.	They must imitate the movements of the teacher	Imitation.	We will stand in a semicircle, with the teacher standing in front of them.	Wide room, without obstacles of vision or furniture.	Five minutes	CD and music equipment.
Draw	We draw the perceived emotion while listening to the work corresponding to the emotion.	A sheet of paper will be handed out to each of them, where they will have to make a drawing according to the emotion they perceive.	Draw according to the emotion you feel.	Seated.	Wide room, without obstacles of vision or furniture.	Ten minutes.	Paper, colours, pencils, chairs, tables, CDs, and music equipment.
Tell a tale	Narration of the story selected for the session	Without indicating what a story is going to be told, we will start directly with the narration of the story, adjusting the timbre of the voice to the different characters that appear.	We will involve the participants in the narration through gestures and onomatopoeia.	We will involve the participants in the narration through gestures and onomatopoeia.	Wide room, without obstacles of vision or furniture.	Ten minutes.	Tale
Sing	Assembly of the song selected for the session, by repetition. We will first focus on the chorus. The verses will be sung by the teacher and they will intervene with the onomatopoeias used in the story.	We will stand in front of the teacher to follow her directions	Repetition.	We will stand in a semicircle, with the teacher standing in front of them.	Wide room, without obstacles of vision or furniture.	Fifteen minutes.	None.
Assembly	We ask different questions related to the story that has been read in order to know which emotions they have identified.	Intervention guidelines will be given to the group. Questions are asked to the group, hoping that they will spontaneously answer. If no one intervenes, one of the participants is appointed to start the conversation	Creation of a relaxed atmosphere that favors a greater degree of participation.	Participants will continue to be seated.	Wide room, without obstacles of vision or furniture.	Ten minutes.	None.
We accompany	Accompanying the work selected for the session with instruments from the environment.	They will be seated, differentiated into two or three groups. Each one of them will be assigned an instrument from the environment (table, chairs, and paper).	Follow the instructions given by the teacher.	Sitting in three different groups.	Wide room, without obstacles of vision.	Five minutes.	Chairs, tables, paper, CDs, and music equipment.
Movement	Free movement through space on the rhythm of the selected song.	They will be sitting, walking, or standing, individually or in groups.	Follow the instructions given by the teacher.	Free	Wide room, without obstacles of vision.	Five minutes.	Chairs, CDs, and music equipment.
We say goodbye	Dance the song “Corazón” [25] Standing in front of the teacher, as they listen to the song they will imitate her gestures and movements.	They must imitate the movements of the teacher	Imitation.	We will stand in a semicircle, with the teacher standing in front of them.	Wide room, without obstacles of vision or furniture.	Five minutes.	CD and music equipment.

**Table 3:** Enjoy the emotions by participants.

	Session 1	Session 2	Session 3	Session 4	Session 5	Session 6	Session 7	Session 8
Participant 1	1	1	1.3	0	1.3	2.16	2.16	2.16
Participant 2	1.5	1.8	0	0	1.8	2	3	3
Participant 3	1	1.8	1.6	1.6	1.6	3	3	3
Participant 4	1	0	1.8	0	0	2	3	3
Participant 5	0.83	1	1.8	1.6	1.8	2	3	2.8
Participant 6	2	1.16	2	2	0	3	3	3
Participant 7	2	2	2	0	3	3	3	3
Participant 8	1.16	2	2	2	2	3	3	3

## Data Availability

Research data are currently available on the website, while the ministerial repository publishes the research data.
